# The Impact of Menopause on Cardiovascular Aging: A Comprehensive Review of Androgen Influences

**DOI:** 10.7759/cureus.43569

**Published:** 2023-08-16

**Authors:** Aditya Raj, Swarupa Chakole, Suyash Agrawal, Anannya Gupta, Harshal Khekade, Roshan Prasad, Tejaswee Lohakare, Mayur Wanjari

**Affiliations:** 1 Community Medicine, Jawaharlal Nehru Medical College, Datta Meghe Institute of Higher Education and Research, Wardha, IND; 2 Medicine, Jawaharlal Nehru Medical College, Datta Meghe Institute of Higher Education and Research, Wardha, IND; 3 Internal Medicine, Jawaharlal Nehru Medical College, Datta Meghe Institute of Higher Education and Research, Wardha, IND; 4 Pediatrics, Smt. Radhikabai Meghe Memorial College of Nursing, Wardha, IND; 5 Research and Development, Jawaharlal Nehru Medical College, Datta Meghe Institute of Higher Education and Research, Wardha, IND

**Keywords:** cardiovascular outcomes, precision medicine, risk factors, hormone replacement therapy, androgens, cardiovascular aging, menopause

## Abstract

Menopause represents a critical life stage in women, characterized by hormonal changes that significantly impact cardiovascular health. While the decline in estrogen levels has long been recognized as a major contributor to cardiovascular aging in menopausal women, the role of androgens, particularly testosterone, has gained increasing attention in recent years. This comprehensive review aims to provide a thorough understanding of the impact of menopause on cardiovascular aging, with a specific focus on the influences of androgens.

A literature search was conducted to gather relevant studies and clinical evidence exploring the relationship between menopause, androgens, and cardiovascular health. The review integrates findings from various studies to present a holistic view of the topic.

The review outlines the changes in hormone levels during menopause and discusses the cardiovascular risk factors associated with this transition. Furthermore, it explores the impact of menopause on cardiovascular structure and function, elucidating the underlying mechanisms that contribute to cardiovascular aging. Androgens' significance in maintaining cardiovascular homeostasis is discussed, followed by exploring the effects of androgen decline during menopause on lipid profiles, insulin sensitivity, vascular function, and other cardiovascular parameters. The review delves into the mechanisms of androgen action on the cardiovascular system, emphasizing the role of androgen receptors and the intricate interplay between androgens, estrogens, and other hormones. Clinical evidence supporting the effects of androgens on cardiovascular aging is presented, including studies investigating the association between androgen levels and cardiovascular outcomes. Additionally, the impact of androgen replacement therapy (ART) on cardiovascular risk markers and events in menopausal women is examined, along with controversies and conflicting findings surrounding the use of androgen therapy in cardiovascular aging.

This structured review provides a comprehensive understanding of the impact of menopause on cardiovascular aging, with a specific focus on the role of androgens. By highlighting the significance of androgens in cardiovascular health during menopause, this review aims to create an initial impression and interest among readers, inviting potential citations in the future. The findings underscore the need for further research and offer insights into managing cardiovascular aging in menopausal women, including lifestyle interventions, pharmacological approaches, and the potential role of personalized medicine and precision therapies.

## Introduction and background

Menopause is a natural biological process that marks the end of a woman's reproductive years, typically around 45-55. It is characterized by the cessation of menstruation and a decline in hormone levels, particularly estrogen and progesterone. Cardiovascular ageing refers to the structural and functional changes in the cardiovascular system as individuals age. These changes can include alterations in blood vessels, cardiac function, and overall cardiovascular health [1,2].

Menopause is a critical life stage for women and is associated with various physiological and hormonal changes. These changes have been linked to an increased risk of cardiovascular diseases like coronary artery disease, heart failure, and stroke. It is crucial to understand the impact of menopause on cardiovascular health to develop effective strategies for the prevention, early detection, and management of cardiovascular diseases in menopausal women. By comprehensively reviewing the literature, we can gain insights into the complex relationship between menopause and cardiovascular ageing, improving patient care and outcomes [3,4].

The purpose of this review article is to provide a comprehensive analysis of the impact of menopause on cardiovascular ageing, with a specific focus on the influences of androgens. While the decline in estrogen levels has traditionally received significant attention in the context of menopause and cardiovascular health, emerging evidence suggests that androgens, such as testosterone, may also play a role. By examining the effects of androgen decline during menopause and the potential mechanisms of androgen action on the cardiovascular system, this review aims to broaden our understanding of the interplay between hormones and cardiovascular ageing. Furthermore, we will evaluate the clinical evidence regarding androgen replacement therapy and its potential benefits for cardiovascular health in menopausal women. Ultimately, this review article seeks to provide valuable insights into the complex relationship between menopause, androgen influences, and cardiovascular ageing, thereby guiding future research and clinical practice in this field.

## Review

Methodology

**Figure 1:**
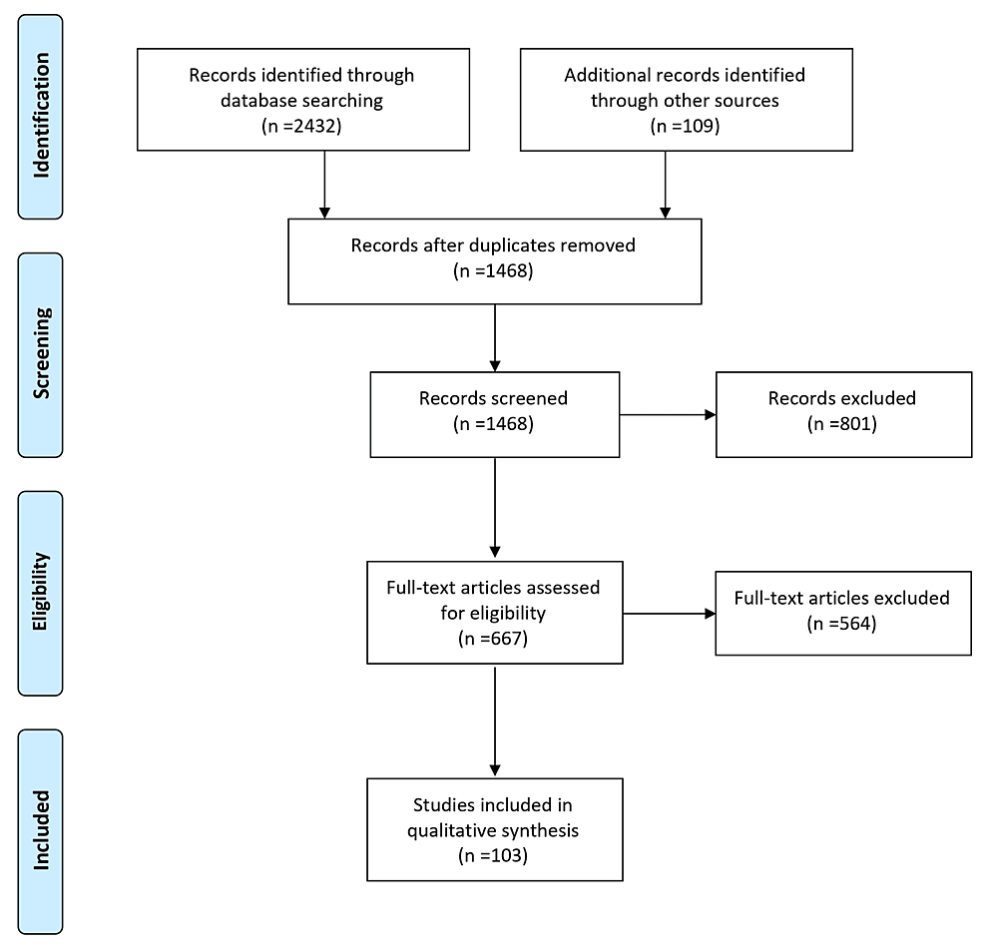
The selection process of articles used in this study. Adopted from the Preferred Reporting Items for Systematic Reviews and Meta-Analyses (PRISMA).

A comprehensive literature search strategy was implemented to identify relevant studies investigating the impact of menopause on cardiovascular ageing and the influences of androgens. Electronic databases, including PubMed/MEDLINE, Embase, and Google Scholar, were searched using keywords and MeSH terms. The search was limited to articles published within the last 10 years without language restrictions. The reference lists of relevant articles and review papers were manually screened to identify additional studies. To ensure the inclusion of appropriate studies, specific inclusion criteria were applied. Eligible studies needed to focus on the impact of menopause on cardiovascular ageing and the influences of androgens involving menopausal women as the study population, clinical trials, and observational studies. Studies unrelated to the research question, those focusing on non-human subjects or in vitro/experimental models, and articles lacking sufficient data or available only as conference abstracts, editorials, commentaries, or opinion articles were excluded. The selection process involved screening the titles and abstracts of identified articles, followed by a full-text assessment based on the inclusion and exclusion criteria. Any disagreements or uncertainties were resolved through discussions among the authors. By implementing these rigorous methodology steps, we aimed to ensure the inclusion of high-quality and relevant studies in this review article, thus providing a comprehensive and reliable overview of the impact of menopause on cardiovascular ageing and the influences of androgens. Figure 1 describes the selection process of articles used in our study.

Menopause and cardiovascular ageing

Changes in Hormone Levels During Menopause

Menopause is characterized by a significant decline in ovarian hormone production, particularly estrogen and progesterone. Estrogen, in particular, plays a crucial role in maintaining cardiovascular health in premenopausal women. During menopause, a marked decrease in circulating estrogen levels leads to hormonal imbalance and subsequent physiological changes within the cardiovascular system [5,6].

Estrogen decline: Estrogen plays a crucial role in maintaining cardiovascular health, exerting various cardioprotective effects. Firstly, estrogen promotes vasodilation by enhancing the production of nitric oxide and prostacyclin, leading to improved endothelial function and increased blood flow. Secondly, estrogen contributes to favorable lipid profiles by increasing high-density lipoprotein (HDL) cholesterol levels and reducing low-density lipoprotein (LDL) cholesterol levels. This lipid-modifying effect helps prevent the development of atherosclerosis and reduces the risk of cardiovascular events. Additionally, estrogen has anti-inflammatory properties and can inhibit the expression of adhesion molecules and pro-inflammatory cytokines, thereby attenuating inflammation within the arterial wall. Furthermore, estrogen inhibits smooth muscle cell proliferation and migration, which helps maintain arterial wall integrity and reduces the risk of plaque formation and vessel narrowing. However, the decline in estrogen levels during menopause disrupts these cardioprotective mechanisms, potentially contributing to the increased cardiovascular risk observed in postmenopausal women [7,8].

Progesterone decline: Although progesterone levels decrease to a lesser extent than estrogen during menopause, the decline in progesterone is also noteworthy in cardiovascular health. Progesterone exhibits vasodilatory effects by enhancing nitric oxide production and stimulating the relaxation of blood vessels. This vasodilation contributes to the maintenance of normal blood pressure. Additionally, progesterone has anti-inflammatory effects and can modulate the renin-angiotensin-aldosterone system, which plays a significant role in blood pressure regulation. The decline in progesterone levels during menopause may further exacerbate the cardiovascular changes associated with estrogen deficiency, potentially contributing to increased cardiovascular risk in postmenopausal women [9,10].

Cardiovascular Risk Factors Associated with Menopause

Menopause is associated with several cardiovascular risk factors, some directly influenced by hormonal changes. These risk factors contribute to the development and progression of cardiovascular diseases in menopausal women [11,12].

Dyslipidemia: Menopause is often accompanied by unfavorable changes in lipid profiles, including increased levels of total cholesterol, low-density lipoprotein cholesterol (LDL-C), and triglycerides, as well as decreased levels of high-density lipoprotein cholesterol (HDL-C). These alterations in lipid metabolism contribute to the development of dyslipidemia, a major risk factor for atherosclerosis and cardiovascular events. Elevated LDL-C and triglyceride levels and reduced HDL-C promote plaque formation in arterial walls and impair vascular function [13,14].

Hypertension: The incidence of hypertension tends to increase after menopause. Estrogen protects vascular health by promoting vasodilation, enhancing nitric oxide production, and regulating blood pressure. However, during menopause, estrogen levels decline, leading to a loss of these protective effects. This hormonal imbalance contributes to endothelial dysfunction, arterial stiffness, and an overall increase in blood pressure, thereby increasing the risk of developing hypertension [15,16].

Insulin resistance and diabetes: Menopause is associated with an increased risk of insulin resistance and the development of type 2 diabetes. Estrogen deficiency during menopause affects insulin sensitivity and glucose metabolism. It impairs the body's ability to utilize insulin, leading to insulin resistance effectively. Additionally, estrogen deficiency is linked to alterations in adipokine secretion, such as increased pro-inflammatory adipokines and decreased production of anti-inflammatory adipokines. These changes further contribute to insulin resistance, impaired glucose metabolism, and an increased risk of diabetes and cardiovascular disease [17,18].

Obesity and body composition change: Menopause often coincides with changes in body composition, including an increase in central adiposity (abdominal fat) and a shift towards a higher body fat percentage. Hormonal fluctuations and metabolic alterations during menopause influence these changes. Increased abdominal fat accumulation, particularly visceral adiposity, is associated with metabolic disturbances such as insulin resistance, dyslipidemia, and chronic low-grade inflammation. These metabolic changes, combined with the pro-inflammatory adipokines released by adipose tissue, contribute to an increased risk of cardiovascular disease [19,20].

Impact of Menopause on Cardiovascular Structure and Function

Endothelial dysfunction: Estrogen is crucial in maintaining optimal endothelial function for cardiovascular health. It promotes vasodilation, inhibits inflammation, and prevents oxidative stress within the endothelium. However, the decline in estrogen levels during menopause can lead to endothelial dysfunction. This dysfunction is characterized by reduced nitric oxide bioavailability, impaired vasodilation, increased vasoconstriction, and an enhanced pro-inflammatory and pro-thrombotic state. These changes contribute to the development of atherosclerosis, impaired blood flow regulation, and an increased risk of cardiovascular events [21].

Arterial stiffness: Menopause is associated with increased arterial stiffness, which refers to the loss of elasticity and compliance in the arterial walls. Estrogen deficiency is thought to be a key factor contributing to this arterial stiffening. As estrogen levels decline, the arterial walls become stiffer, leading to pulse wave velocity (PWV) and augmentation index (AI) changes. Increased arterial stiffness strains the heart and blood vessels, resulting in elevated systolic blood pressure, impaired coronary perfusion, and increased cardiovascular workload. These alterations in arterial stiffness contribute to the development and progression of cardiovascular diseases, such as hypertension and heart failure [22,23].

Cardiac remodeling: Postmenopausal women often experience cardiac remodeling, which refers to structural and functional changes in the heart. These changes include alterations in left ventricular structure and function. Estrogen deficiency and hormonal imbalances associated with menopause are believed to play a significant role in cardiac remodeling. Postmenopausal women may exhibit increased left ventricular mass, reduced diastolic function (impairment in the heart's ability to relax and fill with blood during the relaxation phase), and impaired myocardial contractility (reduced ability of the heart muscle to contract effectively). These cardiac structure and function changes increase the risk of heart failure and other cardiovascular complications in menopausal women [24]. Understanding the impact of menopause on cardiovascular structure and function is crucial for identifying individuals at higher risk for cardiovascular disease during this phase of life. Furthermore, these insights can help guide the development of targeted interventions to prevent and manage cardiovascular complications in menopausal women.

Androgen influences on cardiovascular ageing

Overview of Androgens and Their Role in Cardiovascular Health

Androgens, primarily testosterone, are typically associated with male physiology but play a significant role in women's health. Androgens are produced by the ovaries and adrenal glands in women, albeit at lower levels than men. These hormones affect various physiological systems, including cardiovascular [25,26].

Androgen receptors: Androgens affect the cardiovascular system by binding to androgen receptors, expressed in various cardiovascular tissues, including blood vessels, the heart, and cardiac myocytes. These receptors are crucial in mediating androgen signaling pathways and regulating cardiovascular function. Upon binding of androgens, the androgen receptors initiate intracellular signaling cascades that modulate gene expression, protein synthesis, and cellular responses in cardiovascular cells. The activation of androgen receptors by androgens influences processes such as vascular tone, inflammation, oxidative stress, and remodeling, impacting cardiovascular health [27,28].

Androgen metabolism: Androgens undergo metabolism within target tissues, producing other active and inactive metabolites. Enzymes such as aromatase and 5-alpha reductase play key roles in these metabolic pathways. Aromatase converts testosterone to estradiol, an estrogenic hormone, while 5-alpha reductase converts testosterone to dihydrotestosterone (DHT), a potent androgen. The activity of these enzymes influences the balance between androgenic and estrogenic effects within the cardiovascular system. The conversion of testosterone to estradiol through aromatase activity may contribute to the estrogenic effects on cardiovascular function, while the conversion to DHT can modulate androgen receptor activation and signaling. These androgen metabolic pathways further shape the overall effects of androgens on the cardiovascular system and have implications for cardiovascular health and ageing [29,30].

Effects of Androgen Decline during Menopause

During menopause, there is a decline in androgen levels in addition to the well-known decrease in estrogen. This decline in androgens may have significant implications for cardiovascular health in menopausal women [31].

Vascular effects: Androgens play a role in maintaining vascular health by exerting vasodilatory effects and promoting endothelial function. They help regulate vascular tone and contribute to the maintenance of normal blood pressure. However, during menopause, there is a decline in androgen levels, which may lead to endothelial dysfunction, impaired vasodilation, and increased vascular resistance. These changes can contribute to the development of hypertension and other cardiovascular complications [32,33].

Lipid profile: Androgens influence lipid metabolism, regulating cholesterol levels, lipid particle size, and distribution. Higher androgen levels have been associated with favorable lipid profiles, characterized by lower levels of low-density lipoprotein cholesterol (LDL-C) and higher levels of high-density lipoprotein cholesterol (HDL-C). Conversely, the decline in androgen levels during menopause may contribute to unfavorable lipid profile changes, including increased LDL-C and decreased HDL-C levels. These alterations in lipid metabolism can contribute to an increased risk of atherosclerosis and cardiovascular disease [34-36].

Insulin sensitivity: Androgens play a role in modulating insulin sensitivity and glucose metabolism. Higher androgen levels have been associated with improved insulin sensitivity and glucose uptake in peripheral tissues. However, the decline in androgen levels during menopause may contribute to insulin resistance, impaired glucose metabolism, and an increased risk of metabolic disorders like type 2 diabetes. Insulin resistance is a key factor in developing cardiovascular risk factors, including obesity, dyslipidemia, and hypertension, further highlighting the importance of androgen influences on cardiovascular health during menopause [37-39].

Mechanisms of Androgen Action on the Cardiovascular System

The mechanisms by which androgens influence cardiovascular health involve various cellular and molecular pathways. Understanding these mechanisms can provide insights into the role of androgens in cardiovascular ageing [40,41].

Vasodilation and vascular function: Androgens promote vasodilation, which is the widening of blood vessels. This effect occurs through multiple mechanisms, including increased production of endothelial nitric oxide, a key molecule involved in vasodilation. Androgens also modulate endothelin signaling, a pathway that regulates vascular tone, and contribute to regulating smooth muscle tone in blood vessels. By promoting vasodilation, androgens help maintain optimal vascular function and regulate blood pressure [42,43].

Anti-Inflammatory effects: Androgens exhibit anti-inflammatory properties within the cardiovascular system. They have been shown to attenuate the inflammatory response in vascular cells, reducing the expression of pro-inflammatory cytokines. This anti-inflammatory effect is significant because inflammation plays a critical role in the development and progression of atherosclerosis, a condition characterized by plaque buildup in the arteries. By mitigating inflammation, androgens may help protect against the development of atherosclerosis and other inflammatory cardiovascular conditions [44,45].

Cardiac function: Androgens influence various aspects of cardiac function. They can modulate the growth and contractility of cardiomyocytes, the muscle cells responsible for heart contraction. Additionally, androgens may affect calcium handling within cardiomyocytes, which is crucial for proper cardiac function. Furthermore, androgens have been implicated in cardiac remodeling processes, such as hypertrophy (enlargement) and fibrosis (excessive deposition of connective tissue). The specific mechanisms by which androgens exert these effects on cardiac function are still being investigated. It is likely that intracellular signaling pathways and regulation of gene expression play key roles in mediating these influences [46].

Androgen Replacement Therapy and Its Potential Benefits for Cardiovascular Health in Menopausal Women

Androgen replacement therapy (ART) has been explored as a potential intervention to mitigate the cardiovascular effects of androgen decline during menopause. The administration of exogenous androgens to menopausal women aims to restore physiological androgen levels and potentially provide cardiovascular benefits [47,48].

Cardiovascular risk markers: Numerous studies have indicated that androgen replacement therapy (ART) in menopausal women can favor cardiovascular risk markers. These include improvements in lipid profiles, such as decreased levels of total cholesterol, LDL cholesterol, and triglycerides, as well as increased levels of HDL cholesterol, which is considered cardioprotective. Additionally, ART has been associated with enhanced insulin sensitivity, which can help mitigate the risk of developing insulin resistance and metabolic disorders. Furthermore, markers of vascular function, such as endothelial function and arterial stiffness, have shown improvement with ART. These positive changes in cardiovascular risk markers suggest a potential reduction in overall cardiovascular risk among menopausal women undergoing ART [49].

Cardiovascular outcomes: The impact of ART on cardiovascular outcomes in menopausal women remains an area of ongoing research and debate. While some studies have reported potential benefits, such as a reduced risk of cardiovascular events (e.g., myocardial infarction, stroke, cardiovascular mortality), other studies have yielded conflicting or inconclusive results. It is important to note that the available evidence is primarily derived from observational studies and subgroup analyses of large clinical trials. Therefore, the causal relationship between ART and cardiovascular outcomes is not definitively established. Further well-designed, prospective, randomized controlled trials are needed to provide more conclusive evidence regarding the effects of ART on long-term cardiovascular outcomes in menopausal women [50].

Considerations and controversies: The use of ART in menopausal women requires careful consideration due to potential risks and side effects. Androgen therapy has been associated with adverse effects such as acne, hirsutism, voice changes, and, rarely, liver dysfunction. Moreover, concerns have been raised regarding the potential increased risk of hormone-sensitive cancers, such as breast and endometrial cancer, with androgen therapy. However, the available evidence on these risks remains limited and inconclusive. The optimal dosage, duration, and specific patient populations that may benefit the most from ART are still being investigated. Healthcare providers must engage in shared decision-making with menopausal women, weighing the potential benefits against the potential risks and individual patient characteristics, to make informed decisions regarding the use of ART for cardiovascular health [51,52]. Further research is needed to elucidate the precise mechanisms of androgen action on the cardiovascular system and determine ART's safety and efficacy in menopausal women for cardiovascular health. It is important to note that the use of ART should be individualized and guided by healthcare professionals based on a thorough assessment of the patient's overall health and cardiovascular risk profile.

Clinical evidence of androgen effects on cardiovascular ageing

Studies Exploring the Association Between Androgen Levels and Cardiovascular Outcomes

Several clinical studies have investigated the association between androgen levels and cardiovascular outcomes in menopausal women. These studies have explored the relationship between endogenous androgens, cardiovascular events incidence, and the impact of androgen deficiency on cardiovascular risk [53-61].

Androgen levels and cardiovascular risk: Numerous studies have examined the relationship between endogenous androgen levels, such as testosterone, and cardiovascular risk in menopausal women. Some findings have indicated that lower levels of androgens are associated with an increased risk of cardiovascular events, including myocardial infarction and stroke. This suggests that androgens may possess protective effects on cardiovascular health. However, it should be noted that the precise mechanisms underlying this relationship and the optimal androgen levels for cardiovascular protection are still being explored [62].

Hormonal imbalance and atherosclerosis: Hormonal imbalances that occur during menopause, such as a decrease in androgens and an increase in the estrogen-to-androgen ratio, have been linked to the development and progression of atherosclerosis. Atherosclerosis is a key underlying factor in cardiovascular diseases, characterized by plaque buildup in arterial walls. The delicate balance between estrogen and androgens is crucial in maintaining cardiovascular health. An altered estrogen-to-androgen ratio, favoring estrogen dominance, may promote atherosclerotic processes. Conversely, a more balanced hormonal profile, including adequate levels of androgens, may exert protective effects against atherosclerosis and related cardiovascular complications [63].

Impact of Androgen Replacement Therapy on Cardiovascular Risk Markers and Events in Menopausal Women

Clinical trials and observational studies have investigated the effects of androgen replacement therapy (ART) on cardiovascular risk markers and outcomes in menopausal women. These studies aimed to evaluate whether ART could improve cardiovascular health in the context of androgen decline during menopause [64-68].

Lipid profiles: Several studies have provided evidence of favorable changes in lipid profiles following androgen replacement therapy (ART) in menopausal women. These changes include reductions in total cholesterol, LDL-C (low-density lipoprotein cholesterol), triglyceride levels, and improvements in HDL-C (high-density lipoprotein cholesterol) levels. These improvements in lipid profiles may contribute to a more cardioprotective profile and potentially lower the risk of cardiovascular events [6,14,69-73].

Insulin sensitivity and glucose Metabolism: ART has demonstrated potential benefits in improving insulin sensitivity and glucose metabolism in menopausal women. Insulin resistance and impaired glucose metabolism are associated with an increased risk of cardiovascular disease and type 2 diabetes. By enhancing insulin sensitivity and glucose metabolism, ART may help mitigate these risk factors and reduce the overall cardiovascular risk in menopausal women [74].

Vascular function: Studies have suggested that ART can positively affect vascular function in menopausal women. Specifically, ART has been associated with improvements in endothelial-dependent vasodilation, which reflects the ability of blood vessels to dilate in response to increased blood flow. Additionally, ART has been linked to reduced vascular stiffness, an important marker of arterial health. These vascular function improvements may indicate ART's potential protective effect on the cardiovascular system [75].

Cardiovascular events: The impact of ART on cardiovascular events in menopausal women remains a subject of debate and ongoing research. Some studies have reported a potential reduction in cardiovascular events with ART in menopausal women, suggesting a protective effect on the cardiovascular system. However, conflicting results and studies showing no significant effects have also been reported. Further research is needed to elucidate the precise relationship between ART and cardiovascular events, including long-term, large-scale studies and randomized controlled trials [76].

Conflicting Findings and Controversies Surrounding Androgen Therapy in Cardiovascular Ageing

Despite some positive findings, conflicting findings and controversies surround using androgen therapy in cardiovascular ageing. These controversies stem from various factors, including differences in study design, patient populations, and treatment protocols [77].

Heterogeneity of study findings: Studies exploring the effects of androgen therapy on cardiovascular outcomes have yielded diverse findings, contributing to the heterogeneity in the literature. Some studies have reported positive effects on cardiovascular risk markers and events, such as improved lipid profiles, endothelial function, and reduced incidence of cardiovascular events. However, other studies have shown no significant benefits or indicated potential risks associated with androgen therapy. These conflicting findings emphasize the need for further research and larger-scale clinical trials to elucidate the true impact of androgen therapy on cardiovascular health in menopausal women [78].

Potential risks and side effects: Considering the risks and side effects associated with androgen therapy, particularly at higher doses, is essential. Androgen therapy can lead to virilization, characterized by developing masculine characteristics, acne, increased hair growth, and potential adverse effects on liver function. These potential risks and side effects highlight the importance of carefully assessing the balance between potential benefits and risks when considering androgen therapy as a treatment option. Long-term safety data and optimized dosage regimens are necessary to ensure the safe and effective use of androgen therapy for cardiovascular health in menopausal women [79].

Individualized treatment approach: Given the heterogeneity of study findings and potential risks associated with androgen therapy, the decision to utilize this treatment modality should be individualized for each patient. When making treatment decisions, it is crucial to consider the patient's overall health, cardiovascular risk profile, and potential benefits versus risks. Factors such as age, comorbidities, and the presence of specific cardiovascular risk factors should be taken into account. Additionally, the optimal treatment duration and specific patient populations that may benefit the most from androgen therapy require further investigation. Personalized medicine approaches, including genetic and biomarker profiling, may help identify individuals more likely to benefit significantly from androgen therapy while minimizing potential risks [80,81]. Further research is needed to clarify the potential benefits and risks of androgen therapy in cardiovascular ageing and identify specific patient subgroups that may benefit most. Careful consideration and personalized approaches are crucial when considering androgen therapy for cardiovascular health in menopausal women.

Potential mechanisms for androgen effects on cardiovascular ageing

Androgen Receptors and Their Distribution in Cardiovascular Tissues

Androgen receptors (ARs) are widely distributed in various cardiovascular tissues, including blood vessels, heart, and cardiac myocytes. ARs in these tissues suggest that androgens can directly affect cardiovascular health through receptor-mediated signaling [82].

Vascular tissues: Androgen receptors (ARs) are expressed in various components of vascular tissues, including endothelial cells and smooth muscle cells of blood vessels. Activation of ARs in endothelial cells has been found to play a role in regulating vasodilation and endothelial function. Through their interaction with ARs in endothelial cells, Androgens can modulate the production and release of endothelium-derived vasodilators such as nitric oxide (NO), leading to changes in vascular tone. Additionally, AR activation in smooth muscle cells can influence vascular tone and remodeling processes, which are important in maintaining blood vessels' structural integrity and functionality. Androgen-mediated signaling in smooth muscle cells can affect the contraction and relaxation of blood vessels, thereby impacting overall vascular function [83].

Cardiac tissues: ARs are also expressed in cardiac myocytes, the cells responsible for the contraction and relaxation of the heart muscle. This suggests that androgens may have direct effects on cardiac structure and function. Activation of ARs in cardiac myocytes can modulate various aspects of cardiac physiology. For example, androgens can influence contractility, affecting the force and speed of cardiac muscle contraction. They can also impact calcium handling within cardiac myocytes, crucial for proper cardiac excitation-contraction coupling. Moreover, androgens can regulate gene expression in cardiac cells, influencing the synthesis of proteins involved in cardiac structure, metabolism, and signaling pathways. These direct effects of androgens on cardiac tissues highlight their potential influence on cardiac structure, function, and cardiovascular health in menopausal women [84].

Cellular and Molecular Mechanisms Underlying Androgen Influences on Cardiovascular Health

The effects of androgens on cardiovascular health involve complex cellular and molecular mechanisms. These mechanisms can impact various aspects of cardiovascular physiology, including vascular function, inflammation, oxidative stress, and metabolism [85].

Vascular function: Androgens play a role in modulating vascular function through multiple mechanisms. They have been shown to enhance endothelial nitric oxide production, a key molecule involved in vasodilation and maintaining vascular tone. Androgens can also affect the release and activity of vasoactive substances, such as endothelin-1 and prostacyclin, which regulate vascular smooth muscle contraction and relaxation. Furthermore, androgens have been implicated in regulating genes involved in vascular remodeling and inflammation, influencing processes such as endothelial cell proliferation, migration, and extracellular matrix remodeling [86].

Inflammation and oxidative stress: Androgens exhibit anti-inflammatory properties and can modulate the inflammatory response in vascular cells. They can suppress the production of pro-inflammatory cytokines, such as interleukin-6 (IL-6) and tumor necrosis factor-alpha (TNF-α), and inhibit the activation of nuclear factor-kappa B (NF-κB), a key transcription factor involved in the inflammatory cascade. Moreover, androgens have been shown to regulate the expression of antioxidant enzymes, such as superoxide dismutase (SOD) and glutathione peroxidase (GPx), thereby reducing oxidative stress and promoting a more favorable redox balance in the cardiovascular system [87].

Metabolism and lipid homeostasis: Androgens are crucial in regulating lipid metabolism and homeostasis. They can influence the expression of genes involved in lipid synthesis, transport, and metabolism, such as fatty acid synthase (FAS), lipoprotein lipase (LPL), and peroxisome proliferator-activated receptor gamma (PPARγ). Androgens have also been implicated in adipocyte function and distribution, affecting factors such as adipocyte size, adipokine secretion, and visceral adiposity. These actions can contribute to alterations in lipid profiles, including changes in total cholesterol, low-density lipoprotein cholesterol (LDL-C), high-density lipoprotein cholesterol (HDL-C), and triglycerides, as well as body composition changes [88].

Interplay between Androgens, Estrogen, and Other Hormones in Cardiovascular Ageing

Estrogen-androgen balance: The balance between estrogen and androgens is crucial for maintaining cardiovascular health. Estrogen has been traditionally recognized for its beneficial effects on the cardiovascular system, including vasodilation, improvement of lipid profiles, and anti-inflammatory properties. Conversely, Androgens play a complex role in cardiovascular health, with positive and negative effects. Disruptions in the estrogen-androgen balance during menopause, characterized by a relative decrease in estrogen levels and androgen decline, can contribute to cardiovascular changes such as impaired vascular function, altered lipid metabolism, increased inflammation, and oxidative stress [89].

Estrogen-androgen receptor crosstalk: The interaction and crosstalk between estrogen and androgen receptors further contribute to the complex hormonal regulation of cardiovascular health. Estrogen receptors can modulate androgen receptor expression, and vice versa, leading to potential cross-regulation of hormonal effects on cardiovascular tissues. This receptor crosstalk may influence gene expression, signaling pathways, and downstream effects on cardiovascular function and pathology. Understanding the interplay between estrogen and androgen receptors provides insights into the intricate mechanisms through which these hormones exert their cardiovascular effects during menopause [90].

Other hormonal influences: In addition to estrogen and androgens, other hormones play significant roles in cardiovascular health and can interact with estrogen and androgen pathways. Progesterone, insulin, growth hormone, and other hormonal factors can modulate the effects of androgens and estrogens on vascular function, metabolism, and other cardiovascular processes. These interactions can potentially modify the cardiovascular outcomes associated with changes in the estrogen-androgen balance during menopause. Further research is needed to elucidate the specific mechanisms and clinical implications of these hormonal influences on cardiovascular ageing in menopausal women [91]. Understanding the intricate interplay between androgens, estrogen, and other hormones is crucial for comprehending the multifaceted effects of hormonal changes on cardiovascular ageing. Further research is needed to elucidate the specific mechanisms and interactions between these hormones and their impact on cardiovascular health.

Strategies for Managing Cardiovascular Ageing in Menopausal Women

Lifestyle Interventions for Reducing Cardiovascular Risk during Menopause

Healthy diet: Encouraging menopausal women to adopt a balanced and heart-healthy diet, such as the Mediterranean diet, can profoundly impact cardiovascular health. The Mediterranean diet emphasizes consuming fruits, vegetables, whole grains, lean proteins, and healthy fats while limiting processed foods, saturated fats, and added sugars. This dietary approach has been associated with improved lipid profiles, better weight management, and reduced inflammation, all of which contribute to a healthier cardiovascular system [92].

Regular physical activity: Regular physical activity is essential for maintaining cardiovascular fitness and overall well-being in menopausal women. Engaging in aerobic exercises, such as brisk walking, jogging, swimming, or cycling, helps improve cardiovascular function, manage weight, and reduce the risk of chronic conditions like hypertension and diabetes. Strength training and flexibility exercises, such as yoga or Pilates, can further enhance muscle strength and flexibility, improving cardiovascular health [93].

Smoking cessation: Smoking is a major cardiovascular risk factor, and menopausal women who smoke are particularly vulnerable to adverse cardiovascular outcomes. Encouraging smoking cessation is crucial in reducing the risk of cardiovascular events. Healthcare professionals should provide appropriate support, counseling, and access to cessation resources to help menopausal women quit smoking successfully. Quitting smoking improves cardiovascular health and has numerous other health benefits [94].

Stress management: Chronic stress has a detrimental impact on cardiovascular health and can contribute to the development and progression of cardiovascular diseases. Menopausal women often experience increased stress due to various factors, including hormonal changes and life transitions. Promoting stress management techniques, such as mindfulness meditation, relaxation exercises, deep breathing techniques, and engaging in enjoyable activities, can help reduce stress levels and improve overall well-being. Managing stress effectively supports cardiovascular health and may also positively influence other aspects of menopausal women's health [95].

Pharmacological Approaches to Promote Cardiovascular Health in Menopausal Women

Hormone replacement therapy (HRT): Hormone replacement therapy involves estrogen and, in some cases, progestogens to manage menopausal symptoms. In addition to symptom relief, HRT may offer cardiovascular benefits. It has been associated with improvements in lipid profiles, endothelial function, and vasomotor tone. However, the decision to use HRT should be individualized, considering the patient's overall health, cardiovascular risk profile, and potential risks associated with HRT, such as an increased risk of breast cancer and thromboembolic events. Close monitoring and regular reevaluation of the benefits and risks are essential [96].

Lipid-lowering medications: Dyslipidemia is a common cardiovascular risk factor in menopausal women. Statins and other lipid-lowering medications may be prescribed to manage dyslipidemia and reduce cardiovascular risk. These medications help lower LDL cholesterol levels, improve lipid profiles, and potentially decrease the risk of cardiovascular events. The choice of lipid-lowering agents should consider individual patient characteristics, including baseline lipid levels, comorbidities, and risk assessment [97].

Antihypertensive medications: Hypertension is another significant cardiovascular risk factor in menopausal women. Antihypertensive medications may be prescribed to manage elevated blood pressure and reduce the risk of cardiovascular events. Commonly used antihypertensive medications include ACE inhibitors, beta-blockers, calcium channel blockers, and diuretics. The selection of specific agents should be based on individual patient characteristics, including blood pressure targets, comorbidities, and tolerability [98].

Antiplatelet therapy: Menopausal women with established cardiovascular disease or those at high risk of cardiovascular events may benefit from antiplatelet therapy. Low-dose aspirin is the most commonly prescribed antiplatelet medication. The decision to use antiplatelet therapy should be based on individual patient characteristics, including the presence of atherosclerotic disease, risk assessment, and a careful evaluation of the potential benefits versus the risks, such as an increased risk of bleeding [99].

Role of Personalized Medicine and Precision Therapies in Managing Cardiovascular Ageing

Genetic and biomarker Testing: Genetic testing and biomarker profiling have emerged as valuable tools in cardiovascular ageing management. Genetic testing can identify genetic variations associated with increased cardiovascular risk, allowing for targeted interventions and personalized treatment strategies. Biomarker profiling involves the measurement of specific molecules or substances in the blood or tissues that can indicate disease presence or progression. Healthcare professionals can gain insights into their cardiovascular risk factors and potential response to specific therapies by assessing an individual's genetic predisposition and biomarker profile. This information can guide treatment decisions and help identify individuals who may benefit from targeted interventions tailored to their unique genetic and biomarker profiles [100].

Novel therapies and targeted interventions: Recent advancements in research and technology have opened up new possibilities for managing cardiovascular ageing in menopausal women. Novel therapies are being developed, targeting specific molecular pathways in cardiovascular disease progression. These may include specific drug targets or gene therapies to address the underlying mechanisms contributing to cardiovascular ageing. Additionally, emerging technologies such as nanomedicine offer promising approaches for delivering therapies with greater precision and efficacy. These innovations aim to provide more effective and personalized treatments, taking into account the individual characteristics and needs of menopausal women [101].

Risk stratification models: Accurate models are crucial in identifying menopausal women at higher cardiovascular risk and tailoring appropriate interventions. These models consider various factors, including age, sex, biomarkers, imaging techniques, and clinical parameters, to assess an individual's risk profile. By incorporating multiple risk factors, these models provide a comprehensive evaluation of cardiovascular health and assist healthcare professionals in prioritizing interventions for those most likely to benefit. Improved risk stratification can optimize treatment decisions, allowing for more targeted and effective interventions in menopausal women, ultimately leading to better cardiovascular outcomes [102].

Incorporating personalized medicine and precision therapies into clinical practice has the potential to optimize the management of cardiovascular ageing in menopausal women, allowing for more tailored and effective approaches to cardiovascular health. However, further research and validation of these approaches are needed to ensure their efficacy and safety in diverse populations [103].

## Conclusions

In conclusion, this comprehensive review has shed light on the impact of menopause on cardiovascular ageing, with a particular focus on the influences of androgens. The findings highlight the significant role of hormonal changes during menopause in shaping cardiovascular health. The decline in androgen levels and estrogen changes can contribute to cardiovascular risk factors, structural alterations, and functional impairments. The review has emphasized the importance of understanding androgen influences on cardiovascular ageing, as they play a multifaceted role in lipid metabolism, vascular function, inflammation, and other key cardiovascular processes. Furthermore, exploring the interplay between androgens, estrogens, and other hormones has revealed the intricate relationships that shape cardiovascular health during the menopausal transition. Although there has been progress in uncovering the effects of androgens on cardiovascular ageing, there are still important gaps in knowledge that call for further research. Understanding the precise cellular and molecular mechanisms underlying androgen influences and determining the long-term safety and efficacy of androgen replacement therapy are critical areas of investigation.

Additionally, personalized medicine approaches and the development of risk stratification models will enhance the ability to tailor interventions for individual patients. The implications for clinical practice are significant. Healthcare professionals need to recognize the impact of menopause and androgen decline on cardiovascular health when assessing and managing menopausal women. Collaboration among different specialties and a multidisciplinary approach is essential for providing comprehensive care and optimizing outcomes.
